# An Alignment-Independent Approach for the Study of Viral Sequence Diversity at Any Given Rank of Taxonomy Lineage

**DOI:** 10.3390/biology10090853

**Published:** 2021-08-31

**Authors:** Li Chuin Chong, Wei Lun Lim, Kenneth Hon Kim Ban, Asif M. Khan

**Affiliations:** 1 Centre for Bioinformatics, School of Data Sciences, Perdana University, Kuala Lumpur 50490, Malaysia; lichuinchong@gmail.com; 2 Faculty of Computing and Informatics, Multimedia University, Cyberjaya 63100, Malaysia; lynerlwl@gmail.com; 3 Department of Biochemistry, Yong Loo Lin School of Medicine, National University of Singapore, Singapore 117596, Singapore; bchbhkk@nus.edu.sg; 4 Beykoz Institute of Life Sciences and Biotechnology, Bezmialem Vakif University, Beykoz, 34820 Istanbul, Turkey

**Keywords:** minimal set, alignment independent, alignment-free, sequence diversity, proteome, virus, UNIQmin

## Abstract

**Simple Summary:**

Viral sequence variation can expand the host repertoire, enhance the infection ability, and/or prevent the build-up of a long-term specific immunity by the host. The study of viral diversity is, thus, critical to understand sequence change and its implications for intervention strategies. Typically, these studies are performed using alignment-dependent approaches. However, such an approach becomes limited with increase in sequence diversity. Herein, we present an alignment-free algorithm, implemented as a publicly available tool, UNIQmin, to determine the effective viral sequence diversity at any rank of the viral taxonomy lineage. UNIQmin enables the generation of a minimal set for a given sequence dataset of interest and is applicable to big data, with a reasonable time performance. The minimal set is the smallest possible number of unique sequences required to represent a given peptidome diversity (pool of distinct peptides of a specific length) exhibited by a non-redundant dataset. This compression is possible through the removal of unique sequences that do not contribute effectively to the peptidome diversity pool. The utility of UNIQmin was demonstrated for the species *Dengue virus*, genus *Flavivirus*, family Flaviviridae, and superkingdom Viruses. The concept of a minimal set is generic and thus possibly applicable to both genomic and proteomic data of non-viral, pathogenic microorganisms.

**Abstract:**

The study of viral diversity is imperative in understanding sequence change and its implications for intervention strategies. The widely used alignment-dependent approaches to study viral diversity are limited in their utility as sequence dissimilarity increases, particularly when expanded to the genus or higher ranks of viral species lineage. Herein, we present an alignment-independent algorithm, implemented as a tool, UNIQmin, to determine the effective viral sequence diversity at any rank of the viral taxonomy lineage. This is done by performing an exhaustive search to generate the minimal set of sequences for a given viral non-redundant sequence dataset. The minimal set is comprised of the smallest possible number of unique sequences required to capture the diversity inherent in the complete set of overlapping *k*-mers encoded by all the unique sequences in the given dataset. Such dataset compression is possible through the removal of unique sequences, whose entire repertoire of overlapping *k*-mers can be represented by other sequences, thus rendering them redundant to the collective pool of sequence diversity. A significant reduction, namely ~44%, ~45%, and ~53%, was observed for all reported unique sequences of species *Dengue virus*, genus *Flavivirus*, and family Flaviviridae, respectively, while still capturing the entire repertoire of nonamer (9-mer) viral peptidome diversity present in the initial input dataset. The algorithm is scalable for big data as it was applied to ~2.2 million non-redundant sequences of all reported viruses. UNIQmin is open source and publicly available on GitHub. The concept of a minimal set is generic and, thus, potentially applicable to other pathogenic microorganisms of non-viral origin, such as bacteria.

## 1. Introduction

Infectious diseases caused by viruses are a primary contributor to the global burden of death and disability [1,2]. The world is struggling against viral diseases, with billions of people afflicted annually, even impacting developed and developing countries with improved living conditions. The still ongoing coronavirus disease 2019 (COVID-19) pandemic, for instance, has threatened the global health systems, with a mortality of more than 3.5 million (https://coronavirus.jhu.edu; accessed on 30 May 2021) and no clear indication of when the disease will be brought under control.

Viral sequence variation, even of a single amino acid, can expand the host repertoire, as in the case of zoonotic viruses, or enhance the infection ability of a virus [3,4,5]. Sequence change can result in the evasion of host-established immunity and prevent the build-up of a long-term specific immunity [6]. This thus poses a challenge in the design of drugs and vaccines against viruses and can require a constant need to keep up with the evolving diversity [7,8,9,10]. The effect of viral antigenic diversity on vaccine efficacy and the need to keep up is well-demonstrated for influenza A virus (IAV), where annual re-formulation has been a recommendation by the World Health Organization (WHO) for decades [11]. Highly effective vaccines or drugs are still not publicly available for many notable viral diseases. The study of viral diversity is, thus, imperative in understanding sequence change and its implications for intervention strategies [9,12,13].

The rapid expansion in public sequence data provides an unprecedented opportunity to study viral adaptation and evolution. The National Center for Biotechnology Information (NCBI) public sequence databases consist of ~4.1 M nucleotide and ~10.9 M protein sequences (as of May 2021). Given the importance of viral diversity analyses, various sequence studies have been performed using alignment-dependent approaches [14,15,16,17] that focus on identifying and positioning corresponding regions of individual bases or amino acids. The utility of such an approach, however, is inversely proportional to the increase in sequence diversity, due to the corresponding decrease in conserved regions to anchor the alignment, particularly for highly diverse viruses [18]. This is further limiting when applied to the search for universal vaccine targets that capture the diversity of multiple subtypes or subgroups of a viral species, such as influenza A subtypes or human immunodeficiency virus 1 (HIV-1) clades. Moreover, when expanded to the genus rank, such conserved regions are typically non-existent [19]. Towards this, alignment-free or -independent approaches can offer an alternative to the study of sequence diversity. Such an approach can be defined as a method of quantifying sequence similarity or dissimilarity without the need to use dynamic programming or produce an alignment. Over the past decades, this has been implemented through a variety of methods, which can be mainly grouped into word frequency methods and those that do not resolve the sequence with fixed word-length segments [18].

**Figure 1:**
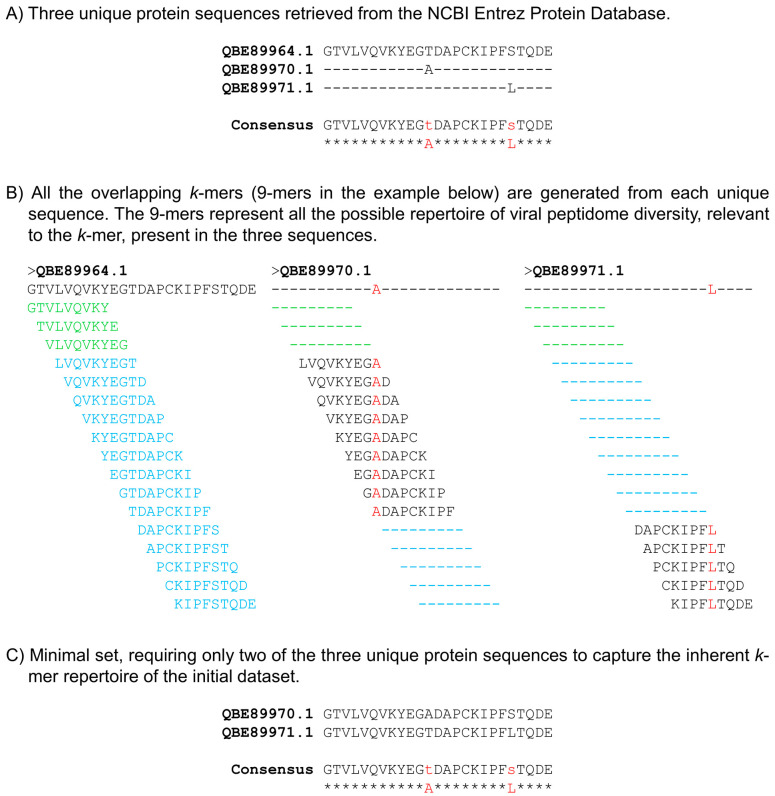
Definition of a minimal set. (**A**) Three unique dengue virus envelope protein sequences (QBE89964.1, QBE89970.1, and QBE89971.1) retrieved from the NCBI Entrez Protein Database are shown aligned (only a 25 amino acids fragment is shown for demonstration purposes). The consensus of the alignment is shown, with positions of variability indicated by the small case residues (variable amino acids A and L colored in red). (**B**) All the overlapping *k*-mers (9-mers in this example) are generated from each unique sequence. The 9-mers represent all the possible repertoire of viral peptidome diversity, relevant to the *k*-mer, present in the three sequences. Although these three sequences are each unique, they share identical 9-mers. The 9-mers shown in green color are those that are identical in all the three sequences, while those in cyan are identical between two of the sequences. The unique 9-mers are shown in black color with variable residues indicated in red. All the 9-mers in sequence QBE89964.1 have a match in at least one of the other two sequences; thus, the *k*-mer repertoire of this sequence can be collectively covered by the other two sequences, rendering the sequence QBE89964.1 as redundant. (**C**) Minimal set, requiring only two of the three unique protein sequences (QBE89970.1 and QBE89971.1) to capture the inherent *k*-mer repertoire of all the unique sequences in the initial dataset.

Previously, Khan et al. (2005) described an alignment-independent method that performs an exhaustive search to determine the minimal set of sequences for a given dataset [20,21]. The minimal set herein refers to the smallest possible number of unique sequences required to represent the diversity inherent in the entire repertoire of overlapping *k*-mers encoded by all the unique sequences in the given dataset. Such dataset compression is possible through the removal of unique sequences, whose entire repertoire of overlapping *k*-mer(s) can be represented by other sequences, thus rendering them redundant to the collective pool of sequence diversity. Applied to a protein dataset for the study of amino acid substitutions, the complete set of peptides of a given *k*-mer length, encoded in the dataset, can be referred to as part of the viral peptidome [22]. The concept of a minimal sequence set is illustrated in Figure 1. Briefly, a given non-redundant (nr) dataset of unique sequences can possess a repertoire of *k*-mers that represents the inherent viral peptidome diversity (for the said *k*-mer length), which can be collectively covered by a smaller fraction of the unique sequences in the initial input dataset; this smaller fraction is termed as the minimal set. The study of a minimal set has thus far been reported for only two viruses, specifically at the species rank, namely *Dengue virus* (DENV) [20,21] and *Influenza A virus* (IAV) [23], which provided important insights into effective sequence diversity and evolution of the two viruses. Thus, this merits further exploration for other viruses, not just at the species rank, but at any rank of the viral taxonomy lineage, such as genus, family or even at the highest, superkingdom rank (all reported “Viruses”).

The reported algorithm by Khan et al. [20,21] is not scalable for large datasets at higher taxonomic lineages and thus requires optimization with regards to (i) the demand on the computational resource, (ii) optimality of the minimal set generated, and (iii) redundancies in the algorithm. To address these issues, we derived a novel algorithm that is significantly improved and scalable for massive datasets, such as all reported viral sequences in nature. The alignment-independent algorithm has also been implemented as a tool, UNIQmin, to allow for a user-specific search of the minimal set. The tool is open source and publicly available at https://github.com/ChongLC/MinimalSetofViralPeptidome-UNIQmin (accessed on 25 August 2021). We describe herein, the algorithm, the tool, its performance evaluation, and application to the study of viral diversity.

## 2. Materials and Methods

### 2.1. UNIQmin—Algorithm

**Figure 2:**
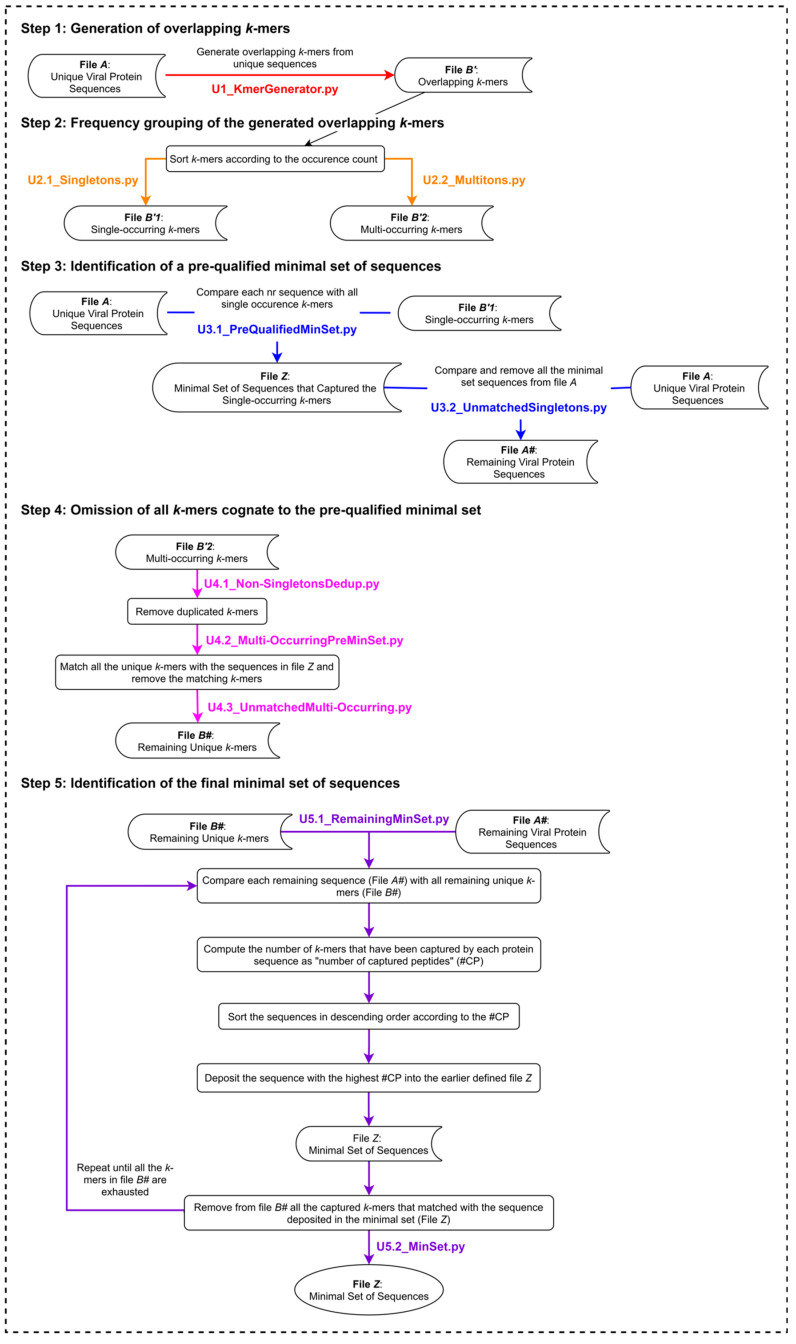
The UNIQmin algorithm. Abbreviation: nr, non-redundant.

In general, the algorithm is comprised of the following five steps: (i) generation of overlapping (sliding) *k*-mers of length *k*, (ii) frequency grouping of the generated overlapping *k*-mers, (iii) identification of a pre-qualified minimal set of sequences, (iv) omission of all *k*-mers cognate to the pre-qualified minimal set, and (v) identification of the final minimal set of sequences. The algorithm is detailed in Figure 2.

Briefly, UNIQmin accepts an input file containing a set of nr protein sequences (referred to as file *A*, in FASTA format) to generate a set of overlapping *k*-mers (referred to as file *B’*) from each sequence. The nr input file can be generated by the use of the clustering tool, Cluster Database at High Identity with Tolerance (CD-HIT) [24] on the redundant dataset of interest, retrieved from a database or prepared in-house. Users may consider removing sequences that contain an unknown residue (denoted with X) to avoid a possibly inflated minimal set. The overlapping *k*-mers (file *B’*) are then grouped according to their frequency (occurrence) count, into either a file containing single-occurring (referred to as *B’1*) or multi-occurring (referred to as *B’2*) *k*-mers. All single-occurring *k*-mers of *B’1* are then matched with each sequence of file *A* to identify the sequences that captured or contained the respective *k*-mers; such sequences qualify as members of the minimal set and thus are subsequently deposited into the minimal set file *Z*. These pre-qualified minimal set sequences are also then removed from the input file *A*, thus resulting in a working input file that would only contain the remaining sequences, referred to as *A#*. The multi-occurring *k*-mers of file *B’2* are removed of duplicates, resulting in only single copy (unique), multi-occurring *k*-mers. The unique, multi-occurring *k*-mers are then matched to *k*-mers of the pre-qualified sequences in the minimal set file *Z*. Matching *k*-mers are removed, resulting in another working file (referred to as *B#*). The remaining unique, multi-occurring *k*-mers of *B#* are matched with those of each remaining sequence of *A#*. A count of this match is stored as the “number of captured peptides” (#CP) for each sequence in *A#*. All sequences of *A#* are then sorted according to the #CP in descending order, from the highest (maximal *k*-mer coverage) to the lowest. This step aims to identify the sequence with the maximal *k*-mer coverage, which is then deposited into the minimal set file *Z*. The deposited sequence and the captured *k*-mers are then removed from files *A#* and *B#*, respectively. This process is repeated until all the remaining *k*-mers in file *B#* are exhausted.

### 2.2. Deployment of UNIQmin

**Scheme 1:**
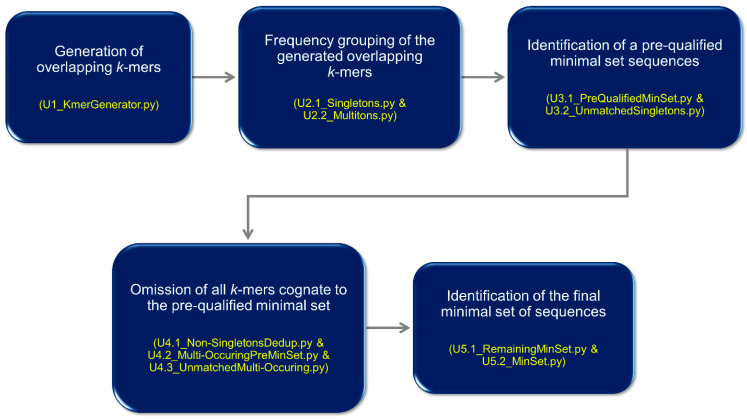
Overview of the UNIQmin tool with the respective Python script(s) employed for the relevant steps of the algorithm indicated.

The different steps of the algorithm were coded by use of the Python programming language (Python 3.7), utilizing multiple packages or modules, including math, pandas, and Biopython, among others. The UNIQmin python scripts used for the respective steps are indicated by name in Figure 2 and Scheme 1. The scripts can be executed individually or as a single pipeline shell script, provided as “UNIQmin.sh” on the GitHub repository page. Additional readme instructions on how to use UNIQmin are also made available on the page. A more detailed description of the algorithm and the tool are also available on the UNIQmin GitHub repository page (https://github.com/ChongLC/MinimalSetofViralPeptidome-UNIQmin/blob/master/README.md; accessed on 25 August 2021), with sample input and output files provided.

To achieve a performance speed-up, a multiprocessing function was added in the first script (U1_KmerGenerator) to allow one to accelerate the task based on the number of available central processing unit (CPU) cores. The Python pyahocorasick library, fast and memory-efficient for string search, was also incorporated into the UNIQmin tool, which contributed significantly to the speed-up. The serial processing of the final step (U5.1_RemainingMinSet script), however, does not contribute to the overall speed-up. Nonetheless, collectively, the five-step implementation of the algorithm in UNIQmin showcased scalability to big data.

### 2.3. Determining k-mer Size of Choice for UNIQmin

A key consideration in the use of UNIQmin is the size (length) of the overlapping *k*-mer window. The size choice can affect the number of sequences in the final minimal set, the validity of the set, and the time taken to determine the set. There are several considerations when deciding on the appropriate size of the *k*-mer. Theoretically, the size of the *k*-mer can range from one amino acid to the length of the longest sequence in the nr dataset of interest. The size of one or two (1–2) amino acids is not appropriate given the random nature of such small matches. In such a case, for example, a large dataset of nr sequences can be easily represented by a small minimal set consisting of only a few unique sequences, which is spurious. This is because although the minimal set captures the entire repertoire of the 1- or 2-mers, the *k*-mers do not recapitulate the true diversity of the initial set of nr sequences, as the order of the amino acids in the sequences is not effectively maintained. The widely used similarity search method, Basic Local Alignment Search Tool (BLAST) algorithm accepts a *k*-mer window size of three amino acids [25,26]. Thus, this length can be considered as the smallest acceptable *k*-mer size for biological significance, although the likelihood of random matches remains, which reduces as the *k*-mer size increases. The concept of a minimal set is possibly applicable to genomic data. Given the composition difference to protein data, the default *k*-mer size used by BLAST for nucleotide searches is 11 bases; thus, this may be considered as the smallest acceptable *k*-mer length for genomic data. Separately, the immune system has the unique ability to differentiate “self” (own body cells) from “non-self” (foreign materials, such as cells infected by viruses) at the molecular level [27]. The adaptive cellular immune system discriminates through recognition of peptides of length 8–15 amino acids, bound to human leukocyte antigen (HLA) class I and 12–25 amino acids for HLA class II [28,29,30,31]. The length of nine amino acids is reported to be typical for peptides bound to HLA class I and as the binding core for peptides bound to HLA class II [28,32]. As such, a *k*-mer size of nine may be considered for immunological or biological applications in general [17,33,34,35]; however, emphasis should be given to the research question for the relevance of the *k*-mer size. The length of the longest sequence in the dataset as the largest *k*-mer size that can be used, is not recommended, as there would be no or limited (potentially, if unique partial sequences are present in the dataset) compression of the initial input set. The above are non-empirical considerations on deciding the appropriate *k*-mer size.

**Figure 3:**
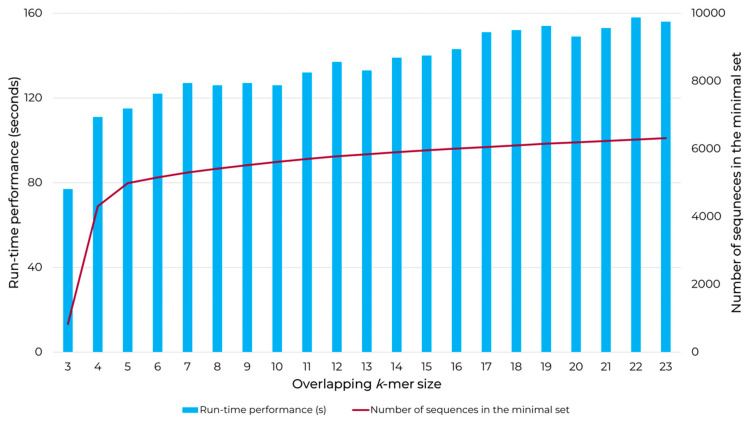
Comparison of the number of sequences in the minimal set and the run-time performance for different overlapping *k*-mer sizes.

The recommended *k*-mer size for a dataset of interest can be determined heuristically through a comparative analysis. This would involve determining the minimal set and also evaluating the performance for different *k*-mer lengths by use of the input nr dataset of interest or its subset (if sufficiently large). Figure 3 provides an illustration of the minimal set and run-time performance for different *k*-mer sizes (3–23 amino acids), obtained from an input nr dataset of 9800 dengue virus protein sequences (see Section 3.1 for details on the dataset). An increasing trend is observed for both run-time performance and the number of sequences in the minimal set with an increase in *k*-mer size. This depicts a decrease in the compression with an increase in *k*-mer size. The higher reduction observed for 3-mer relative to others is indicative of the inherent higher likelihood of randomness. The *k*-mer sizes of the range, from 7 to 10 for the given dataset, appear to provide a good balance when considering the number of sequences in the minimal set and the time taken to determine the set.

Having a statistical measure to determine the optimal *k*-mer size for the minimal set is desired. We had considered the effort by others in similarly addressing the problem of *k*-mer optimization, but for other biological applications, such as genomic sequence assemblies and generating alignment-free dendrograms [36,37,38,39]. Given the various trade-offs in solving biological problems and the difficulty in mathematically quantifying them, an explicit formula for selecting the *k*-mer size, which considers the various effects, has been elusive. It is possible to estimate some acceptable bound or range for the *k*-mer based on certain aspects of the data (for example, genome size and coverage); however, such estimates do not usually account for the impact of some key attributes of the data (such as repetitiveness of the genome, heterozygosity rate or read error rate). In practice, the *k*-mer is often chosen based on previous experience with similar datasets; for a comprehensive evaluation, these efforts may be time-consuming. Despite these challenges, progress has been made in determining the optimal *k*-mer. These attempts appear to rely on an objective function, defining features or aspects of the problem that help indicate the reliability or desirability of the output. Even then, these are not devoid of caveats. In the study of the minimal set herein, a general objective function that can help assess the desirability of one minimal set over the other is hard to define (considering possible loss of biological signal and other caveats—see the last two paragraphs of the Discussion section). In summary, identifying the optimal *k*-mer is challenging, and a range of *k*-mer values may be considered, based on heuristic evaluations, in line with the research question. The problem of determining the optimal *k*-mer in the search for the minimal set merits further investigation as a study by itself.

### 2.4. Application of UNIQmin—Data Retrieval, Processing, and Data Analysis across Viral Taxonomic Lineages (Species, Genus, and Family Ranks)

All reported protein sequences of DENV were retrieved from the NCBI Entrez Protein Database using taxonomy identifier (txid) of “12637” (as of March 2019). The retrieved sequence data (redundant dataset) was deduplicated of identical sequences using the clustering tool, CD-HIT [24], resulting in an nr DENV protein dataset. UNIQmin was then applied to the nr dataset to generate a minimal set of the viral peptidome. A *k*-mer window size of nine (9; nonamer) was used to evaluate the viral peptidome diversity with respect to the cellular immune response (antigenic diversity) [21,33,34,40,41]. Further, to expand the analysis to higher ranks of taxonomic lineages, all reported protein sequences of DENV genus and family ranks were retrieved from the NCBI Entrez Protein Database (as of December 2019). Similarly, the retrieved datasets were then removed of duplicates, and the deduplicated datasets were used for the generation of the minimal sequence sets using UNIQmin.

### 2.5. Performance Comparison with Other Existing Alignment Independent Data Compression Methods

The DENV nr dataset (Section 2.4) was used for performance comparison analysis between ITERmin, a re-implementation herein of the earlier Khan algorithm (KA) [20,21], and UNIQmin. Additionally, a literature search showed that several protein compressors have been developed [42,43], which, however, mainly focus on the reduction of sequence file size for storage purposes. Nonetheless, a comparison was performed against the existing compressors to evaluate the compression ability of UNIQmin. Towards this, all reported protein sequences of *Homo sapiens* (HS) were retrieved from the NCBI Entrez Protein Database by use of the txid “9606” (as of March 2020). An HS dataset was used because it was the only one that allowed both direct and an approximate indirect (with 2019 HS dataset) comparison between the compressors. Direct comparison of UNIQmin was only carried out with Gzip (www.gzip.org, accessed on 3 March 2020), a widely used compressor that balances speed and compression [43], and AC, which typically showcases the best compression [42]. The direct comparison between UNIQmin, Gzip, and AC was made by use of the 2020 HS dataset (HS^2020^) and 2018 all reported viral sequence dataset (“All viruses”), retrieved from the NCBI Entrez Protein Database by use of the txid “10239” (as of November 2018), while indirect comparisons were made with other tools using earlier HS datasets (HS^2019^) and viral datasets of *Acanthamoeba polyphaga* (AP^2019^) and *Enterococcus phage* (EP^2019^) [42].

## 3. Results

### 3.1. Application of UNIQmin—Dengue Virus (DENV) Lineage as a Usage Scenario

A total of 26,205 DENV protein sequences (as of March 2019) were retrieved from the NCBI Entrez Protein Database. Removal of duplicates by use of CD-HIT returned a total of 9800 nr DENV protein sequences (a reduction of ~62.6% from the redundant dataset), which was used as an input dataset to the UNIQmin tool. UNIQmin first generated a set of 17,328,090 overlapping 9-mers from the input dataset, which were categorized according to the occurrence count. Approximately ~0.5% (82,566) of the 9-mers were singletons (single-occurring) and ~99.5% (17,245,524) were multi-occurring. A total of 5048 sequences were required to capture all the single-occurring 9-mers. These sequences also captured the majority of multi-occurring 9-mers (17,241,370), leaving behind 4154 nonamers, which were captured by additional 471 sequences. This resulted in a minimal set containing 5519 protein sequences, which is a compression of ~43.7% from the input nr dataset.

We then expanded this approach to higher ranks of the DENV taxonomic lineage, such as the genus *Flavivirus* and family Flaviviridae. As of December 2019, a total of 45,593 and 273,463 protein sequences of genus *Flavivirus* and family Flaviviridae were retrieved, respectively. The sequences from the genus *Flavivirus* dataset were deduplicated, resulting in 17,771 nr sequences with a reduction of ~61.0% from the redundant dataset. After implementing the compression using UNIQmin, there were only 9763 sequences in the minimal set, achieving a compression of ~45.1% from the input nr dataset. The deduplication of the family Flaviviridae sequences resulted in a dataset containing 141,200 nr sequences, which was a drop of ~48.4% from the retrieved redundant sequences. A compression of ~52.8% from the input nr dataset was achieved at the family rank using UNIQmin, providing a minimal set of 66,707 sequences. A summary of UNIQmin compression at the species, genus, and family ranks is shown in Table 1.

### 3.2. Comparison to Existing Methods

A performance comparison between ITERmin and UNIQmin is shown in Table 2, using the DENV nr protein dataset. A detailed comparison between the two algorithms is provided on the GitHub repository page (https://github.com/ChongLC/MinimalSetofViralPeptidome-ITERmin; accessed on 25 August 2021). UNIQmin showed an improvement in performance relative to ITERmin, in terms of removal of more of the unique sequences redundant to the peptidome, which increased from ~43.5% to ~43.7% (15 additional sequences); the difference is expected to be significantly higher for large datasets. More importantly, UNIQmin offered a drastic speed-up of ~273-fold compared to ITERmin for 1000 sequences, which increased to more than 97 k-fold for close to 10 k sequences. This showcased the scalability potential of the tool for application to big data. Demonstrating this, UNIQmin took ~17 days to generate the minimal set for a large dataset of all reported viral sequences (~4.9 M as of November 2018, retrieved from NCBI Entrez Protein Database using the txid “10239”, with 2.2 M non-redundant sequences after deduplication). This was achieved by executing UNIQmin on a machine equipped with Intel(R) Xeon(R) E5-2690 v2 @ 3.00 GHz 40-core processors (only 14-cores were utilized for the first step of each tool, ITERmin and UNIQmin, while subsequent steps utilized one-core), 396 GB of RAM, and 44 TB of local storage.

Generally, the efficiency of compression is calculated in terms of bit-rate (also known as bit per symbol; bps) for performance comparison purposes [42]. The lower the bps, the better the compression. The bit-rate calculation was carried out herein for UNIQmin and compared with six other existing compressors, both directly and indirectly (Table 3). Direct comparison of the six existing tools (not including UNIQmin) showed that Gzip exhibited the worst compression for three of the datasets (HS^2019^, AP^2019^, and EP^2019^), while AC showed the best for dataset HS^2019^, was a close second to the best, Paq81 for dataset AP^2019^, and fourth (relative to the best, Paq81) for dataset EP^2019^. A direct comparison of UNIQmin to AC and Gzip (HS^2020^ and “All viruses” datasets) showed that AC provided the best compression, followed by Gzip. Hosseini et al. (2019) reported AC as the best compressor amongst the existing tools/approaches in terms of bps, while Gzip appeared to be the best when memory usage and time were considered. In comparison, UNIQmin trailed behind Gzip in terms of compression; however, it should be noted that UNIQmin focuses on identifying the minimal set of unique sequences while maintaining the total repertoire of the relevant *k*-mer peptidome diversity, whereas the others do not involve direct removal of sequences. This means that the compression ability of UNIQmin is directly proportional to the conservation level of the sequences within the dataset. Thus, UNIQmin offers an alternative to the existing compression methods by focusing on the removal of unique sequences that are degenerate or redundant to the peptidome.

## 4. Discussion

The idea of a minimal set, herein, is essentially a compression problem [42,44,45], applied to the study of viral protein sequence diversity, without incurring any loss of information in terms of the total peptidome repertoire (relevant to the *k*-mer of choice). Hence, the minimal set is the smallest fraction of the non-redundant protein sequences required to represent the complete peptidome repertoire present in the dataset (relevant to the *k*-mer). Thus, the minimal set can be considered to provide insight into the effective sequence diversity and evolution of the virus. The tool, UNIQmin, provides an alternative approach to analyze sequence diversity, commonly done using alignment-dependent approaches, which would be ineffective for the study of a diverse protein sequence dataset. A diverse dataset can be of a highly diverse virus species, such as *Human immunodeficiency virus 1* (HIV-1), or spanning multiple species at higher taxonomic lineage ranks, such as genus or family. Thus, the approach herein may represent a major paradigm shift, as a direct enabler of novel applications in the study of sequence diversity and indirectly contributing to alignment-independent research. For example, the minimal set can be generated spatio-temporally to allow for comparative analyses of sequence diversity. Any decrease in compression relative to a referenced dataset of unique sequences would be indicative of an increase in the repertoire of novel *k*-mers in the collective pool of sequence diversity, while an increase would imply higher *k*-mer redundancy relative to the referenced dataset. Moreover, the minimal set can be subjected to further downstream analyses, coupled with alignment-dependent approaches, where applicable. It is expected that over time, the minimal set would show limited growth with increases in sequence data for a given taxonomy rank, assuming a balanced and sufficient sampling. This is because as the number of non-redundant sequences grows, viral variants exhibiting *k*-mers with novel sequence change, relative to the existing pool of sequence diversity, become less likely, and even if observed, such viral variants would be limited in number; unless the pool is perturbed by sequencing of previously unknown members of the taxonomy rank.

The UNIQmin algorithm is a significant improvement from that of the Khan algorithm (KA) and one that offers the best combination of speed and optimality of the minimal set generated. It was observed to be 263-fold faster than ITERmin, a re-implementation of KA. UNIQmin achieved the speed-up performance by recognizing and taking advantage of the fact that singleton *k*-mers only occur once in the dataset. Thus, the sequences from which they originated are already candidates for the minimal set. This avoids not only the need to process those sequences but also eliminates the need to evaluate multi-occurring *k*-mers that are also present within the singleton-*k*-mer-harboring sequences. This pre-filtering, utilizing single-occurring *k*-mers, ended up capturing ~91.5% (5048 sequences) of the eventual 5519 dengue virus minimal set sequences (derived from a starting input dataset of 9800 nr DENV sequences), prior to the execution of the canonical core steps of the algorithm for the remaining sequences. More importantly, UNIQmin was scalable for application to big data. It was successfully applied to ~2.2 M nr protein sequences of all reported viruses, deduplicated from ~4.9 M downloaded sequences (retrieved as of November 2018), the number of which has now grown to ~9.9 M (as of May 2021), with ~3.3 M nr sequences. UNIQmin is expected to still manage well this increase in data, with a better speed-up performance utilizing machines with a larger number (>14) of CPU cores. We also demonstrated the utility of UNIQmin in compressing diverse datasets spanning lineage ranks, such as *Flavivirus* (genus) and Flaviviridae (family). The resulting minimal sets covered the proteomic diversity within (intra) and between (inter) the viral species members of the genus or family. Additionally, UNIQmin demonstrated a reasonable compression in bit-rate relative to other well-known tools (Gzip and AC), with compression directly proportional to sequence conservation within the dataset, without impacting the inherent diversity.

There are caveats applicable to UNIQmin. Primarily, it is the reliance on the input sequence order (for the canonical part of the algorithm), when two or more sequences share the highest #CP (the highest number of *k*-mers matched or “captured” by a sequence relative to the repertoire of the *k*-mers available; see step 5 of Figure 2). Ideally, each sequence choice should be simulated to determine which would result in a more optimal minimal set. This was not explored because preliminary simulations with a decision tree approach (data not shown) indicated that this would be computationally intensive (in the quest to exhaust the *k*-mer library), as two or more sequences having the highest #CP was a common observation. This meant that not only multiple decision tree simulations would be required for a given input sequence, but that each decision point simulation may also end up requiring its own simulation (i.e., simulation within the simulation). It should be noted that this is only an issue when two or more sequences with the highest #CP share a dissimilar list of matched *k*-mers. When an identical list of matched *k*-mers is shared, the selection of one sequence as part of the minimal set would render the other with zero matches to the pool of remaining *k*-mers (file *B#*) in the subsequent iteration of comparisons. Such sequences (with zero matches) should be removed, which will provide a significant speed-up, and this will be considered as a future improvement of UNIQmin.

Another caveat is the possibility of potentially losing relevant biological signals. For example, the sequences of an nr dataset that are not selected as part of the minimal set may contain biologically relevant signals that are missing from the minimal set. Figure 1 can be used to illustrate this. The sequence QBE89964.1 is not included as part of the minimal set because it does not contribute any unique 9-mers relative to the other two sequences in the initial nr dataset. However, this would not be true for a *k*-mer size of 10 amino acids, as the sequence would end up contributing the unique 10-mer, “TDAPCKIPFS”. If this peptide is biologically relevant, then this signal would be lost for a minimal set defined for *k*-mer of size nine. Thus, the spread of the substitutions in the sequence can impact the effectiveness of the *k*-mer size in capturing biological signals. Another factor that can influence signal loss is the size of the nr dataset. Such a signal is likely to be diluted or lost in big data, where the compression is expected to be much higher. For example, UNIQmin compressed the Flaviviridae nr dataset of 141,200 sequences by ~52.8% (Table 1). A reduction of more than half of the nr sequences would suggest possible loss of biological signals present in the sequences removed. There is an expected increase in data size with ascending increase in hierarchy of ranks across the viral taxonomic lineage, such as from species to family rank. Thus, this caveat may be more pertinent at higher taxonomic ranks, such as family, order, class, and phylum, among others. Nonetheless, despite the possible loss of biological signals, the minimal set serves as a representative of the nr dataset, with much-reduced data size and is applicable to any rank of the viral taxonomy lineage. Towards this, we compared alignment-based phylogenetic trees generated using an nr dataset of dengue virus NS2B protein and its minimal set (data not shown). The phylogenetic tree derived from 155 nr sequences was well represented by a tree derived from its minimal set of 99 sequences, preserving the topology of the major clades. The application of the minimal set for downstream analyses and the possible effect of loss of biological signals merits further investigation.

## 5. Conclusions

UNIQmin enables the generation of a minimal set for a given sequence dataset of interest, without the need for sequence alignment. This alignment-independent approach allows one to select data that spans various ranks of the taxonomic lineage and provides the opportunity to ask relevant research questions with respect to effective sequence diversity, which would not be possible by the use of alignment-dependent approaches. Notably, it is also able to reduce big data size approximately by half, which is welcome, even though computing power is becoming cheap and more pervasive. This enables the exploration of big data compression prior to diversity or related analyses. This would allow for more efficient use of computer resources and would be a boon to those who have limited access to a big data computer infrastructure. The concept of a minimal set is generic and thus possibly applicable to both genomic and proteomic data of other pathogenic microorganisms of non-viral origin, which generally exhibit much larger data size, such as archaea (~7.0 M protein sequences as of May 2021), bacteria (~798 M), and Eukaryota (~108 M).

## Figures and Tables

**Table 1:** A summary of compression using UNIQmin across the viral lineages ranks, namely species, genus, and family.

Taxonomic Lineage Rank ^#^	Number of Retrieved Sequences	Number of nr Sequences ^#^| Percentage of Deduplication (Relative to the Retrieved Sequences)	Number of Sequences in the Minimal Set	Compression Using UNIQmin (Relative to the Retrieved Sequences|nr Dataset) ^^^
Species: *Dengue virus*	26,205	9800|~62.6%	5519	~16.3%|~43.7%
Genus: *Flavivirus*	45,593	17,771|~61.0%	9763	~17.6%|~45.1%
Family: Flaviviridae	273,463	141,200|~48.4%	66,707	~27.2%|~52.8%

^^^ Reported values are rounded to one decimal place. ^#^ Abbreviations: nr, non-redundant.

**Table 2:** Performance evaluation of ITERmin and UNIQmin in generating a minimal set for dengue virus protein sequences.

Input Dataset (Number of nr Sequences) ^#^	Measure	Algorithm Implementation *	
		ITERmin	UNIQmin
1000 sequences **	Run-time performance (minutes)	273	<1 (14 s)
	Number of sequences in the minimal set	851	851
	Compression ^^^	~14.9%	~14.9%
9800 sequences	Run-time performance (minutes)	~194,400	>2 (127 s)
	Number of sequences in the minimal set	5534	5519
	Compression ^^^	~43.5%	~43.7%

* The same server environment was used for the comparison of ITERmin and UNIQmin. ^^^ Reported values are rounded to one decimal place. ^#^ Abbreviation: nr, non-redundant. ** This smaller-scale initial input dataset for ITERmin and UNIQmin was derived through random selection of sequences from the dengue virus nr dataset of 9800 protein sequences.

**Table 3:** Direct and indirect comparisons of the compression performance, in terms of bit-rate (bits per symbol; bps), between UNIQmin, Gzip (a widely used compressor that balances speed and compression), AC (typically showcases the best compression), and other existing protein compressors.

Compressors	Human		Viruses		
	HS^2019^	HS^2020^	AP^2019^	EP^2019^	All Viruses ^^^
Gzip ^#^	4.61	1.55	4.59	4.69	1.61
bzip2 ^#^	4.26	-	4.27	4.49	-
7zip ^#^	4.03	-	4.14	4.59	-
Lzma ^#^	4.03	-	4.14	4.43	-
Paq81 ^#^	3.90	-	3.97	4.30	-
AC ^#,$^	3.79	0.94	3.99	4.52	0.63
UNIQmin	-	1.93	-	-	2.27

^#^ Compressors that were compared by Hosseini et al. (2019). ^$^ Compressor that was developed by Hosseini et al. (2019). ^^^ Dataset retrieved as of November 2018. Abbreviations: HS, *Homo sapiens*; AP, *Acanthamoeba polyphaga*; EP, *Enterococcus phage*.

## Data Availability

Public data was used. All codes and sample input and output files are publicly available from the GitHub repository page (https://github.com/ChongLC/MinimalSetofViralPeptidome-UNIQmin; accessed on 25 August 2021).

## Abbreviations

COVID-19, coronavirus disease 19; IAV, influenza A virus; WHO, World Health Organization; NCBI, National Centre for Biotechnology Information; HIV-1, human immunodeficiency virus 1; DENV, dengue virus; nr, non-redundant; #CP, number of captured peptides; CPU, central processing unit; txid, taxonomy identifier; BLAST, basic local alignment search tool; HLA, human leukocyte antigen; KA, Khan algorithm; HS, *Homo sapiens*; AP, *Acanthamoeba polyphaga*; EP, *Enterococcus phage*; bps, bit per symbol.

## References

[1] Keni R., Alexander A., Nayak P.G., Mudgal J., Nandakumar K. (2020). COVID-19: Emergence, Spread, Possible Treatments, and Global Burden. Front. Public Health.

[2] GBD 2019 Diseases and Injuries Collaborator (2020). Global burden of 369 diseases and injuries in 204 countries and territories, 1990–2019: A systematic analysis for the Global Burden of Disease Study 2019. Lancet.

[3] Steinhauer D.A. (2013). Pathways to human adaptation. Nature.

[4] Wendel I., Matrosovich M., Klenk H.D. (2015). SnapShot: Evolution of Human Influenza A Viruses. Cell Host Microbe.

[5] Thakur A., Mikkelsen H., Jungersen G. (2019). Intracellular Pathogens: Host Immunity and Microbial Persistence Strategies. J. Immunol. Res..

[6] Volkov I., Pepin K.M., Lloyd-Smith J.O., Banavar J.R., Grenfell B.T. (2010). Synthesizing within-host and population-level selective pressures on viral populations: The impact of adaptive immunity on viral immune escape. J. R. Soc. Interface.

[7] Heiny A.T., Miotto O., Srinivasan K.N., Khan A.M., Zhang G.L., Brusic V., Tan T.W., August J.T. (2007). Evolutionarily Conserved Protein Sequences of Influenza A Viruses, Avian and Human, as Vaccine Targets. PLoS ONE.

[8] Khan A.M., Miotto O., Nascimento E.J.M., Srinivasan K.N., Heiny A.T., Zhang G.L., Marques E., Tan T.W., Brusic V., Salmon J. (2008). Conservation and Variability of Dengue Virus Proteins: Implications for Vaccine Design. PLOS Negl. Trop. Dis..

[9] Bingham R.J., Dykeman E.C., Twarock R. (2017). RNA Virus Evolution via a Quasispecies-Based Model Reveals a Drug Target with a High Barrier to Resistance. Viruses.

[10] Chong L.C., Khan A.M. (2019). Identification of highly conserved, serotype-specific dengue virus sequences: Implications for vaccine design. BMC Genom..

[11] Regional Planning (1999). Influenza Pandemic Plan. The Role of WHO and Guidelines for National and Regional Planning.

[12] Raman H.S.A., Tan S., August J.T., Khan M.A. (2020). Dynamics of Influenza A (H5N1) virus protein sequence diversity. PeerJ.

[13] Hackbart M., Deng X., Baker S.C. (2020). Coronavirus endoribonuclease targets viral polyuridine sequences to evade activating host sensors. Proc. Natl. Acad. Sci. USA.

[14] Wolf Y.I., Kazlauskas D., Iranzo J., Lucía-Sanz A., Kuhn J.H., Krupovic M., Dolja V.V., Koonin E.V. (2018). Origins and Evolution of the Global RNA Virome. mBio.

[15] Yang O.O., Ali A., Kasahara N., Faure-Kumar E., Bae J.Y., Picker L.J., Park H. (2015). Short Conserved Sequences of HIV-1 Are Highly Immunogenic and Shift Immunodominance. J. Virol..

[16] Koo Q.Y., Khan A.M., Jung K.-O., Ramdas S., Miotto O., Tan T.W., Brusic V., Salmon J., August J.T. (2009). Conservation and Variability of West Nile Virus Proteins. PLoS ONE.

[17] Yang O.O. (2009). Candidate Vaccine Sequences to Represent Intra- and Inter-Clade HIV-1 Variation. PLoS ONE.

[18] Zielezinski A., Vinga S., Almeida J., Karlowski W.M. (2017). Alignment-free sequence comparison: Benefits, applications, and tools. Genome Biol..

[19] Chong L.C., Khan A.M. (2019). Vaccine Target Discovery. Encyclopedia of Bioinformatics and Computational Biology.

[20] Khan A.M. (2005). Mapping Targets of Immune Responses in Complete Dengue Viral Genomes. Master’s Thesis.

[21] Khan A.M., Heiny A.T., Lee K.X., Srinivasan K.N., Tan T.W., August J.T., Brusic V. (2006). Large-scale analysis of antigenic diversity of T-cell epitopes in dengue virus. BMC Bioinform..

[22] Özer O., Lenz T.L. (2021). Unique Pathogen Peptidomes Facilitate Pathogen-Specific Selection and Specialization of MHC Alleles. Mol. Biol. Evolution..

[23] Heiny A.T. (2005). The Antigenic Diversity Analysis of Complete Viral Genome of Influenza A Virus. Bachelor’s Thesis.

[24] Li W., Godzik A. (2006). Cd-hit: A fast program for clustering and comparing large sets of protein or nucleotide sequences. Bioinformatics.

[25] Altschul S.F., Gish W., Miller W., Myers E.W., Lipman D.J. (1990). Basic local alignment search tool. J. Mol. Biol..

[26] Mahram A., Herbordt M.C. (2010). Fast and accurate NCBI BLASTP: Acceleration with multiphase FPGA-based prefiltering. Proceedings of the 24th ACM International Conference on Supercomputing—ICS’10.

[27] Nicholson L.B. (2016). The immune system. Essays Biochem..

[28] Trolle T., McMurtrey C.P., Sidney J., Bardet W., Osborn S.C., Kaever T., Sette A., Hildebrand W.H., Nielsen M., Peters B. (2016). The Length Distribution of Class I–Restricted T Cell Epitopes Is Determined by Both Peptide Supply and MHC Allele–Specific Binding Preference. J. Immunol..

[29] Gfeller D., Guillaume P., Michaux J., Pak H.-S., Daniel R.T., Racle J., Coukos G., Bassani-Sternberg M. (2018). The Length Distribution and Multiple Specificity of Naturally Presented HLA-I Ligands. J. Immunol..

[30] Sanchez-Trincado J.L., Gomez-Perosanz M., Reche P.A. (2017). Fundamentals and Methods for T- and B-Cell Epitope Prediction. J. Immunol. Res..

[31] Wieczorek M., Abualrous E.T., Sticht J., Álvaro-Benito M., Stolzenberg S., Noé F., Freund C. (2017). Major Histocompatibility Complex (MHC) Class I and MHC Class II Proteins: Conformational Plasticity in Antigen Presentation. Front. Immunol..

[32] EL-Manzalawy Y., Honavar V. (2013). Major Histocompatibility Complex (MHC), Binder Prediction. Encyclopedia of Systems Biology.

[33] Lim W.C., Khan A.M. (2018). Mapping HLA-A2, -A3 and -B7 supertype-restricted T-cell epitopes in the ebolavirus proteome. BMC Genom..

[34] Hu Y., Tan P.T., Tan T.W., August J.T., Khan A.M. (2013). Dissecting the Dynamics of HIV-1 Protein Sequence Diversity. PLoS ONE.

[35] Tan S., Sjaugi M., Fong S., Chong L., Raman H.A., Mohamed N.N., August J., Khan A. (2021). Avian Influenza H7N9 Virus Adaptation to Human Hosts. Viruses.

[36] Pornputtapong N., Acheampong D.A., Patumcharoenpol P., Jenjaroenpun P., Wongsurawat T., Jun S.-R., Yongkiettrakul S., Chokesajjawatee N., Nookaew I. (2020). KITSUNE: A Tool for Identifying Empirically Optimal K-mer Length for Alignment-Free Phylogenomic Analysis. Front. Bioeng. Biotechnol..

[37] Zhang Q., Jun S.-R., Leuze M., Ussery D., Nookaew I. (2017). Viral Phylogenomics Using an Alignment-Free Method: A Three-Step Approach to Determine Optimal Length of k-mer. Sci. Rep..

[38] Cha S., McK Bird D. (2016). Optimizing k-mer size using a variant grid search to enhance de novo genome assembly. Bioinformation.

[39] Chikhi R., Medvedev P. (2014). Informed and automated k-mer size selection for genome assembly. Bioinformation.

[40] Khan A.M., Hu Y., Miotto O., Thevasagayam N.M., Sukumaran R., Raman H.S.A., Brusic V., Tan T.W., August J.T. (2017). Analysis of viral diversity for vaccine target discovery. BMC Med. Genom..

[41] Oliveira S.C., de Magalhães M.T.Q., Homan E.J. (2020). Immunoinformatic Analysis of SARS-CoV-2 Nucleocapsid Protein and Identification of COVID-19 Vaccine Targets. Front. Immunol..

[42] Hosseini M., Pratas D., Pinho A.J. (2019). AC: A Compression Tool for Amino Acid Sequences. Interdiscip. Sci. Comput. Life Sci..

[43] Kryukov K., Ueda M.T., Nakagawa S., Imanishi T. (2020). Sequence Compression Benchmark (SCB) database—A comprehensive evaluation of reference-free compressors for FASTA-formatted sequences. GigaScience.

[44] Hategan A., Tabus I. Protein is compressible. Proceedings of the 6th Nordic Signal Processing Symposium—NORSIG 2004.

[45] Adjeroh D., Nan F. On Compressibility of Protein Sequences. Proceedings of the Data Compression Conference (DCC’06).

